# Resilience against the pandemic: The impact of COVID-19 on migration and household welfare in Tajikistan

**DOI:** 10.1371/journal.pone.0257469

**Published:** 2021-09-20

**Authors:** Satoshi Shimizutani, Eiji Yamada

**Affiliations:** JICA Ogata Sadako Research Institute for Peace and Development, Tokyo, Japan; The Bucharest University of Economic Studies, ROMANIA

## Abstract

The COVID-19 pandemic is likely to have adverse effects on the economy through damage to migration and remittances. We use a unique monthly household panel dataset that covers the period both before and after the outbreak to examine the impacts of COVID-19 on a variety of household welfare outcomes in Tajikistan, where remittance inflows in recent years have exceeded a quarter of annual GDP. We provide several findings. First, after April 2020, the adverse effects of the pandemic on household welfare were significantly observed and were particularly pronounced in the second quarter of 2020. Second, in contrast to expectation, the pandemic had a sharp but only transitory effect on the stock of migrants working abroad in the spring. Some expected migrants were forced to remain in their home country during the border closures, while some incumbent migrants expecting to return were unable to do so and remained employed in their destination countries. Both departures and returns started to increase again from summer. Employment and remittances of the migrants quickly recovered to levels seen in previous years after a sharp decline in April and May. Third, regression analyses reveal that both migration and remittances have helped to mitigate the adverse economic outcomes at home during the “with-COVID-19” period, suggesting that they served as a form of insurance. Overall, the unfavorable effects of the COVID-19 pandemic were severe and temporary right after the outbreak, but households with migrants were more resilient against the pandemic.

## Introduction

This study provides evidence on the impact of the Coronavirus disease 19 (COVID-19) pandemic on migration and household welfare in Tajikistan by using a unique high-frequency household panel dataset that covers the period both before and after the outbreak. By doing so, we aim to inform academics and policymakers on how the pandemic has affected households in a remittance-dependent country.

The COVID-19 pandemic has affected households all over the world in various ways. Adverse effects such as limited mobility and economic recession are not confined within national borders but are likely to spill over to other countries. If we limit our scope to developing countries, a major channel of international transmission of the pandemic is relevant to remittance inflows, since remittances sent by international migrants are now the largest source of external financing for developing countries, exceeding the amount of official development assistance (ODA) and foreign direct investment (FDI) [1]. The pandemic is potentially devastating to those countries because of economic downturns in destination countries that are now under lockdown and suffering from oil price crashes, restrictions on remittances under stringent movement bans, and the cancelation of planned migration [2]. Indeed, remittance inflows to developing countries started to decline after the outbreak in several countries [3]. In April, the World Bank released a pessimistic estimate that remittances to low- and middle-income countries were projected to fall by 19.7% on average, with the largest at 27.5% in Europe and Central Asia by region [4]. Under these circumstances, migrants and their families were regarded as one of the most vulnerable groups among developing countries due to the worldwide economic crisis following the onset of the pandemic [5].

**Fig 1:**
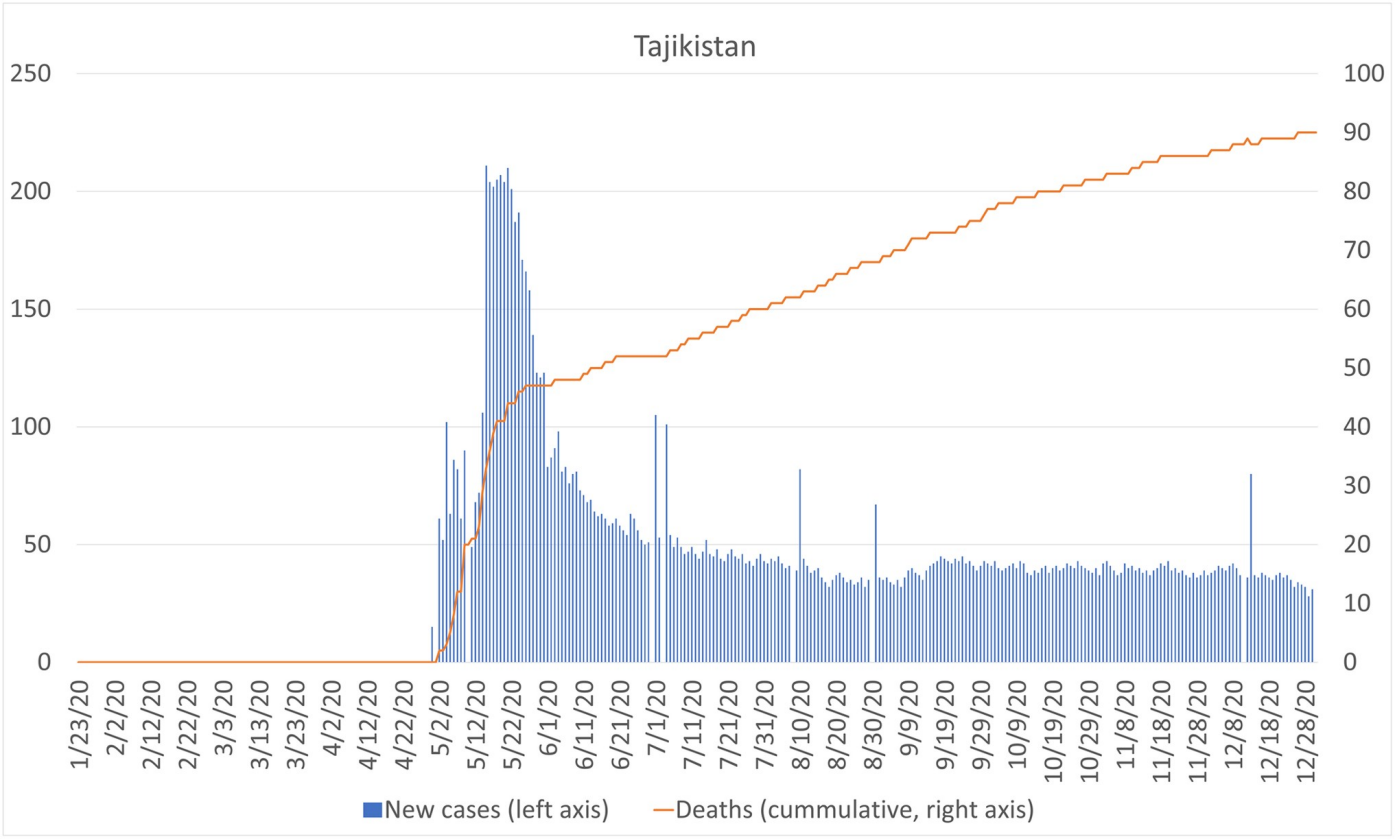
Number of newly confirmed cases and deaths in Tajikistan.

In this study, we examine the case of Tajikistan. Tajikistan is one of the most remittance-dependent countries in the world in terms of remittance inflows relative to GDP, which were estimated to be 28.2% in 2019, making it the fifth-ranked country in the world. In Tajikistan, more than 40% of households have at least one international migrant, and most migrants from Tajikistan are working-age young men residing in rural areas without a job before leaving the country [6]. Given the high prevalence of migration, a substantial decline in remittance inflows may be devastating to household welfare in Tajikistan if the COVID-19 pandemic has indeed caused damage to international migration and remittances. The first case of COVID-19 in Tajikistan was officially declared on April 30, 2020, much later than in other countries. Tajikistan was reluctant to take strong measures to prevent the pandemic, imposing less strict restrictions on movement across international borders and lockdowns in cities. Tajikistan started to close its borders to neighboring countries in March, but rather than imposing a total lockdown, the country pursued a more relaxed approach, even after the first case was confirmed at the end of April (Fig 1). At the end of December 2020, the cumulative number of confirmed cases was 13,296 and the number of reported deaths was 90, much smaller than other Central Asian countries. Given these numbers, which are considered to have been underestimated, the adverse effects of the COVID-19 pandemic seem to have been less serious in Tajikistan, and the country has largely succeeded in containing the pandemic compared with other countries.

However, this is not the end of the COVID-19 pandemic story in Tajikistan, since the main destination country of migrants is seriously affected. Tajik international migrants are highly concentrated in Russia because Tajikistan has maintained close economic ties with Russia as a former Soviet Republic in Central Asia [6]. In 2018, more than 90% of the Tajik migrants headed to Russia to work as marginal laborers in the construction and service sectors [6], suggesting that Tajik labor migrants are low-skilled workers vulnerable to changes in the Russian economy and migration policy.

**Fig 2:**
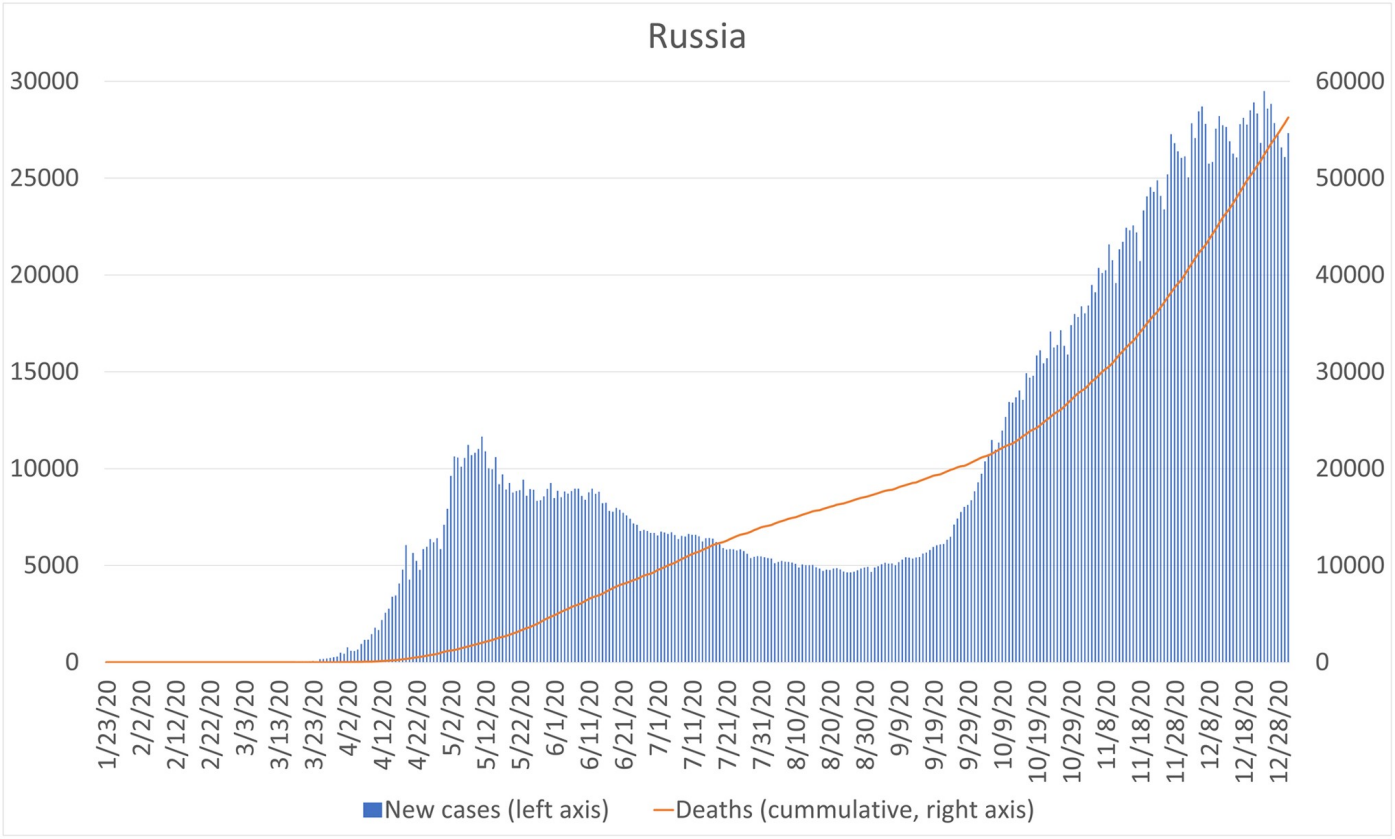
Number of newly confirmed cases and deaths in Russia. Source: Novel Coronavirus (COVID-19) Case Data https://data.humdata.org/dataset/novel-coronavirus-2019-ncov-cases.

In Russia, the first case of COVID-19 was confirmed on January 31, three months before Tajikistan. The number of confirmed cases started to increase beginning in March and the first death was reported in mid-March. The country closed its international borders to foreigners on March 18. In contrast to Tajikistan, the Russian government has imposed a variety of strict measures, such as closing public institutions and canceling events, as well as instituting lockdowns in many large cities. Meanwhile, the number of new confirmed cases per day expanded and the cumulative number of confirmed cases was 3,127,347 as of the end of December (Fig 2).

In this study, we provide evidence of the impact of the COVID-19 on migration and household welfare using a dataset covering both before and after the pandemic up to December 2020. To our knowledge, there has been little research on the impact of the pandemic in the post-COVID period (see the next section for a literature review). Existing papers commonly focused on the very early impact of COVID-19 in spring, finding sharp and adverse effects on household income and food security. Building on this research, we contribute to both policy debates and academic literature in the following ways.

First, our study is empowered by a unique high-frequency household panel dataset that covers the period both before and after the outbreak began. The distinct advantage of the dataset is the monthly collection of data to cover the “with-COVID-19” period up to December 2020. This allows us to explore the impact of the COVID-19 on a variety of outcomes over a longer timeframe than previous studies. To our knowledge, little research examines the effect of the pandemic up to the end of 2020.

Second, we use a monthly household-level dataset in Tajikistan starting in 2015, which means we are able to detect any changes in seasonal patterns of a variety of outcomes caused by the new pandemic. The dataset contains a wide variety of variables related to household welfare, enabling us to explore comprehensive impacts of the pandemic at the household level, and the high frequency improves the efficiency of econometric estimates by capturing larger variations in household behavior.

Third, our dataset is longitudinal, which allows us to take an empirical approach to establish a relationship between remittances and household welfare in a more rigorous way, since longitudinal data enables us to correct unobserved factors and address endogeneity using exogenous shocks to households. There have been a variety of studies reporting the positive impact of remittances on household welfare in Vietnam [7, 8], Pakistan [9], Bangladesh [10], Kenya [11], Malawi [12].

However, their empirical methodology is less rigorous, using cross-sectional data, and most of them employ the PSM (propensity score matching) method because of difficulty in finding valid instrumental variables for remittances. Amare and Lena [7] is an exception that employs a fixed effect estimation. Yang [13] examined the effect of international remittances on households using the appreciation of the Philippine peso during the 1997 Asian financial crisis and found that remittances did not have a significant effect on household consumption but positive and a significant impact on capital accumulation, entrepreneurship, and educational spending. Since the outbreak of COVID-19 is exogenous to each household, we can overcome identification issues pointed to in the previous literature.

## Literature review

This paper contributes to two strands of literatures in economics. The first strand is the large volume of empirical studies on the impact of migration and remittances on household welfare in migrant sending countries. Most of those studies have shown that migration contributes to improve the livelihood of household members at home through remittances. Especially, many studies have pointed out the role of migration and remittances as an insurance to various shocks at the household or local levels. For example, Yang and Choi [14] showed that remittances from overseas migrants play a role as insurance for the remaining family in the home country and 60 percent of declines in household income are replaced by an increased remittance flows for households sending migrants in the Philippines. Haliday [15] found that the Salvadorian migrants in the US send more remittance when their home community face negative agricultural shocks. Mohapatra, Joseph, and Ratha [16] reported that remittances from international migrants contribute to better preparedness to natural disasters at home in Bangladesh, Ethiopia, Burkina Faso, and Ghana. Naudé and Bezuidenhout [17] examined how remittances from Sub-Saharan African migrants in Eurozone respond to natural disasters and conflict in their home countries and found that remittances are relatively stable compared to other financial flows to Africa and they do not significantly respond to the incidents of disasters at home, implying that remittances are a source of resilience.

More recent studies established the micro econometric evidence that households with migrants are better off under various situations. Amare and Hohfeld [7] examined the impact of remittances on the poverty reduction measured by household asset growth in the context of rural Vietnam and found that remittances have a positive effect on asset growth and on poverty reductions. Cuong and Linh [8] reported that international remittances increase per capita income and per capita expenditure of the household in Vietnam while it reduces employment of remaining members. Javed, Awan, and Waqas [9] showed that international migration delivers benefits as an increase in various forms of household expenditures in Pakistan. Wadood and Hossain [10] reported the remittance-receiving households are better off in terms of consumption expenditure compared to non-receiving ones in Bangladesh. There are many studies on the related topic in Africa. Jena [11] confirmed that remittances have a positive and significant impact on the household’s physical investments, assuring that migration and remittances make the household better off in the long-run perspective in Kenya. Kangmennaang, Bezner-Kerr, and Luginaah [12] reported that households with migrant are less food insecure and have greater chance of accumulating asset in Malawi. Those existing studies have shown that migration and remittances contribute to improve the livelihood of households in developing countries under normal economic condition. However, there is still a knowledge gap on the role of migration and remittances to secure the livelihood of the households during global serious economic downturn.

The second strand of the literature is the rapidly emerging micro-econometric studies on the socio-economic impact of the COVID-19 pandemic. Early evidence suggested that the economic impact of the pandemic is devastating. For example, Baker et al. [18] and Chen et al. [15] employed high-frequency data to examine the impact on household spending using a dataset covering the period after the outbreak. Baker et al. [18] revealed that the household spending in the U.S. after the outbreak of the pandemic has shown a great surge and drop according to the increase of the cases. Chen et al. [19] reported that the offline consumption in Chinese cities have severely declined, along with the waves of the COVID-19 cases. Kansiime et al. [20] warns of a decline in earnings and increased food insecurity in Kenya and Uganda using data collected up to April 2020. Amare et al. [21] found greater food insecurity in Nigeria using data collected up to May 2020. However, there has been little research on the impact of the pandemic in the post-COVID period that has employed a dataset collected both before and after the outbreak began.

A subset of the literature focuses on the impact of the COVID-19 on international migration. Murakami et al. [22] reported that remittance inflows will decrease by 14–20% due to the pandemic, and household spending per capita will decline by 1–2% in the Philippines using the data collected before the pandemic started. Among the papers that used data after the outbreak stated, Barker et al. [23] used panel data in Bangladesh and Nepal to show a decline in earnings and a greater prevalence of food insecurity among households with migrants up to June 2020. Mobarak and Vernot [24] showed a decline in labor supply in the village, labor migration, remittance earnings, and total incomes in rural Nepal in May 2020. Honorati et al. [25] reported that half of the Armenian workers expecting to migrate were not able to leave for Russia and lost their jobs because of the suspension of construction activities as of June, which is likely to result in reduced remittances. These findings contrast with the traditional view in the economic literature that regards migration and remittances as a form of insurance for households in developing countries [14].

In this study, we utilize a monthly panel dataset covering the period before and after the outbreak of the pandemic to provide a more granular picture on the impact of the COVID-19 pandemic on migration and remittances in developing countries.

## Materials and methods

We use monthly household-level panel data from the Listening to Tajikistan (L2TJK) survey. The data is proprietary and belongs to the World Bank which conducted survey authorized and approved by the Tajikistan Government. The first 30 rounds were compiled by the World Bank between May 2015 and November 2017 and the subsequent rounds have been financed jointly by the World Bank, UNICEF, and the JICA Ogata Research Institute. All rounds of the survey are conducted by phone and cover a wide variety of variables, including migration, income and employment, and the wellbeing and life satisfaction of households. The 31st and subsequent rounds have added more questions related to migration and remittances. The most recent survey month is December 2020, and the dataset thus covers more than half a year after the outbreak in Tajikistan.

The sample of the survey is 800 households that were randomly drawn from a nationally representative survey consisting of 3,000 households in the spring of 2015 conducted on a face-to-face basis. Households were interviewed at 10-day intervals, which changed to two-week intervals after the sixth round of data collection, and one-month intervals since November 2015. Households who refused to participate were replaced with households from the same primary sampling unit (PSU). In each round, the respondents were asked to provide information on a variety of household characteristics and their perception of food security and economic well-being as well as migration and remittances. Note that most of the variables are collected at the household level, not at the individual level. In this study, we use the data collected from January 2018 to December 2020, as most of the main variables of interest have been collected in a consistent way since mid-2017. We define the “without-COVID-19” period as the months before April 2020 and the “with-COVID-19” period as the months since April 2020.

In order to assess the impact of the pandemic on household welfare, we choose six categories of variables to examine: food security, finance for basic needs, healthcare expenditure, employment, subjective economic wellbeing and financial wellbeing. The first three groups are related to household spending on basic needs. Since food security constitutes a basic human need, we examine the variables on whether a household is able to provide enough food for its members, including children. We also investigate basic needs other than food that are essential to daily life: whether a household can pay for utilities, health expenditures, and other basic needs. Regarding household income to measure economic resources, we focus on employment status: whether a household worked for pay in the previous seven days or received a wage in the previous ten days. Note that our data does not include a question on total income due to the limitations of a phone-based survey. The last two categories refer to household perceptions of economic and financial wellbeing. Subjective economic wellbeing includes variables on how a household perceives its economic status or the economic and job situation of the nearby area. Lastly, financial wellbeing refers to a household’s past and future prospects regarding financial conditions, focusing on changes from the past and to the future.

Table 1 reports the summary statistics of the variables used in this study. All variables reported are binary. The first set of variables is related to household migration status. On average, 29.7% of households have at least one migrant member in the survey month and 10.4% on average had received remittances in the previous ten days. 12.4% of households answered that they have at least one migrant household member who currently has a job in the destination country. The remaining rows show the outcomes related to household welfare in the analyses, which are explained above and investigated further in the next section.

**Table 1:** Summary statistics.

VARIABLES	(1)	(2)	(3)
	N	mean	sd
**Migration/Remittance Variables**			
Household has migrant member in this month	73,144	0.297	0.457
Household received some amount of remittance in past 10 days	73,144	0.104	0.305
Migrant member is currently employed in the destination country	60,168	0.124	0.330
**Household Welfare Variables**			
**(1) Food security**			
Able to buy enough food for members for the past month	73,144	0.831	0.375
Not able to buy enough food for the children in the household	62,697	0.069	0.253
**(2) Financing for basic needs**			
Financially unable to pay for utilities for the past month	73,144	0.288	0.453
Borrow any money over the last month to pay for basic needs	73,144	0.260	0.439
**(3) Healthcare**			
Reduced healthcare expenditure	73,144	0.292	0.455
**(4) Employment**			
Did work for pay in previous 7 days	73,144	0.780	0.414
Received wage in previous 10 days	73,144	0.135	0.342
**(5) Subjective economic well-being**			
Perceives own household as poor	65,284	0.363	0.481
Perceives living area’s economic condition is bad	65,284	0.170	0.376
Area’s job situation is bad	65,284	0.457	0.498
**(6) Subjective financial wellbeing**			
Thinking current financial condition is worse compared with previous month	73,144	0.134	0.341
Expecting financial condition gets worse in next month	73,144	0.087	0.281

*Source*: Authors.

In what follows, we take three steps to explore the impact of the pandemic on migration and household welfare in Tajikistan. First, we examine the overall impact of the COVID-19 pandemic on household welfare in Tajikistan. We statistically compare the seasonal trajectory of household welfare in 2020 to that of regular years (i.e., the average of 2018 and 2019). Second, we examine the monthly flow of migrant departures and returns as well as remittances to look into the timing and magnitude of the adverse shock caused by the pandemic. Third, we perform regression analyses to investigate the role of migration and remittances on household welfare during the pandemic. We compare the variables related to household welfare between households, both those with migrants and remittances and those without. We then measure the resilience of households with migrants and remittances since they are considered to be more resilient to the pandemic if they are better off than the households without migrant and remittances.

### The overall impact of the pandemic on the household welfare

This section examines the overall impact of the COVID-19 pandemic on a variety of household welfare outcomes in Tajikistan without distinguishing between households with migrants and remittances and those without. We compare the average of the outcomes between “regular” years without the pandemic and 2020 with the pandemic. We define “regular” years as the average of 2018 and 2019. We limit the regular years to 2018 and 2019 because some variables have been collected only after the middle of 2017. Since the welfare outcomes fluctuate seasonally, we examine a year-on-year change in the average of outcomes in every quarter before and after the pandemic. For example, we compare the average of each outcome in the first quarter (January to March) of 2020 and that of 2018–2019 with a t-test for the difference in the mean. Since the economic restrictions caused by the pandemic started from the second quarter of 2020, we are interested in the pre-pandemic comparison with the second quarter and the subsequent quarters. The monthly average of each outcome is normalized to the change from January of each year in order to eliminate a yearly trend. The normalized monthly average is used to calculate the quarterly average.

Table 2 reports the year-on-year comparison (difference) of the average of each outcome of the corresponding quarter in each column.

**Table 2:** Comparison of household welfare before and after the COVID-19 outbreak.

	First Quarter	Second Quarter	Third Quarter	Fourth Quarter
**Food Security**				
(1) Able to buy enough food	-0.032 ***	-0.100 ***	-0.097 ***	-0.092 ***
(2) Not able to buy enough food for children	0.004	0.043 ***	0.020 ***	0.013 **
**Financing for basic needs**				
(3) Unable to pay for utilities	0.015 *	0.122 ***	0.078 ***	0.024 **
(4) Borrowed money for basic needs	-0.004	0.040 ***	0.013	-0.010
**Healthcare**				
(5) Reduced health expenditure	0.044 ***	0.036 ***	-0.024 **	-0.086 ***
**Employment**				
(6) Did any paid work	-0.004	-0.146 ***	-0.046 ***	-0.047 ***
(7) Received wage	0.005	0.011	0.011	-0.015 *
**Subjective economic wellbeing**				
(8) Perceive own household as poor	-0.004	0.069 ***	-0.013	-0.018
(9) Area’s economic condition is bad	0.007	0.121 ***	0.071 ***	0.129 ***
(10) Area’s job situation is bad	-0.040 ***	0.173 ***	0.191 ***	0.031 **
**Financial Wellbeing**				
(11) Financial condition is worse the past	0.001	0.150 ***	0.082 ***	0.086 ***
(12) Financial condition will become worse	0.002	0.077 ***	0.037 ***	0.029 ***

Note: Difference of the quarterly average of each outcome in 2020 minus that in the same quarter in 2018/19 is reported. Statistical significance by t-test of the difference is reported by the stars.

*** p<0.01,

** p<0.05,

* p<0.1.

#### Food security

The first two rows report the change in the proportion of households that were able to buy enough food for household members in the previous ten days and that were not able to buy enough food for the children in the same period. While we see a statistically significant decline in the share of households able to buy sufficient food for members in the first quarter of 2020, the negative gap widens to minus 0.1 in the second and subsequent quarters, showing that food insecurity was exacerbated in the second quarter of 2020, after the outbreak, and the situation continued until the fourth quarter. This is also the case for the proportion of households unable to buy enough food for the children. The difference in the share became significantly large in the second quarter to 0.043 and then declined slightly in the third and fourth quarters.

#### Finance for basic needs

Row (3) shows the change in the proportion of households financially unable to pay for utilities in the previous ten days. A substantial increase in the proportion by 12.2 percentage points is observed in the second quarter of 2020, but the size of the gap gradually declined in the third and fourth quarters. Row (4) reports that the share of households borrowing any money to pay for basic needs over the previous ten days significantly increased in the second quarter of 2020. By the third quarter, the proportion was returning to the regular pattern.

#### Healthcare expenditure

Row (5) shows the proportion of households in which health expenditure decreased significantly in the first and second quarters of 2020. However, the trend reversed in the third and fourth quarters, meaning that more households increased their healthcare expenditure in the latter half of 2020.

#### Employment

Row (6) shows the change in the proportion of households that did any paid work in the previous seven days. Reflecting the economic downturn during the pandemic, the proportion of households with a paid job significantly decreased in the second quarter of 2020 by 14.6 percentage points and the negative impact on the paid work became milder in the third and fourth quarters. Row (7) shows the proportion of households that received a wage in the previous ten days. In contrast to Row (6), the share of households receiving a wage does not seem to have been negatively affected during the COVID-19 period.

#### Subjective economic wellbeing

Rows (8), (9), and (10) show that the perception of economic conditions deteriorated in the second quarter of 2020. Row (8) reports that the share of households who thought their household was poor surged in the second quarter of 2020, while it returned to the same level as in regular years in subsequent quarters. Rows (9) and (10) show that the pessimistic perceptions of the economic wellbeing of the nearby area are more pronounced than for their own household, which can be seen from rows (9) and (10). Row (9) shows that there was a significant increase of 12.1 percentage points in the proportion of households that believe the current economic conditions in the city or area where they live are bad or very bad in the second quarter of 2020. Row (10) reports a surge in the proportion of households that think now is a bad time to find a job in the city or area where they live today, as it increased by 17.3 percentage points in the second quarter of 2020 and by 19.1 percentage points in the third quarter.

#### Financial wellbeing

Row (11) shows that the proportion of households thinking that the current financial condition was worse than the previous month grew by 15 percentage points in the second quarter of 2020 and by 8.2 percentage points in the third quarter, while there was no statistical difference in the first quarter. Row (12) reports that the proportion of households expecting financial conditions to get worse in the next month surged significantly in the second quarter of 2020, but the magnitude was smaller (7.7 percentage points) than that for the prospect from the past. These observations suggest that, under the pandemic, households felt strong discontent with the current financial situation compared to the past at the early stage of the pandemic. They also had greater anxiety about the future at the onset. These pessimistic views became weaker after the third quarter while they were still significantly more prevalent than in regular years.

These simple calculations show that the impact of the COVID-19 pandemic was temporarily serious in terms of economic measures in the second quarter of 2020, the period immediately after the COVID-19 pandemic started. While households on average emerged from their worst difficulties in subsequent quarters for most of the variables, the situation remained worse in 2020 than in regular years. The exception was for healthcare expenditure, as a lower share of households reduced expenditure in the third and fourth quarters compared to the pattern in the regular years, reflecting the fact that healthcare spending was a prioritized expenditure in the crisis.

We turn to investigate the role of migration and remittances in the pandemic. In the supplementary text in S1 Appendix, we have included a graphical analysis showing the monthly development of the average outcome of each welfare variable by disaggregating households with migrants versus those without migrants. We observe that the households with migrants have been recovering earlier to meet the regular seasonal pattern than households without migrants, which have also been returning to the regular pattern after a delay. We observe a severe drop in household welfare in spring and early summer, as the previous studies confirmed. What is newly found in this study is that household welfare measured in a variety of outcomes recovered to some extent in the autumn and winter. These observations may seem counterintuitive since many of those expecting to migrate were forced to stay in their home country due to travel restrictions and border closures, which would be plausibly detrimental to remittance inflows during this year. In order to understand the mechanism involved, we examine the effect of the COVID-19 pandemic on migration and remittance in the next section.

### Migration and remittances during the pandemic

This section examines the impact of the pandemic on migration and remittances. There have been many anecdotal episodes on the difficulties faced by those planning to migrate in reaching their intended destination, which was especially the case during mid-March 2020, when Russia closed its international borders. We will review the status of international migration in our dataset below.

**Fig 3:**
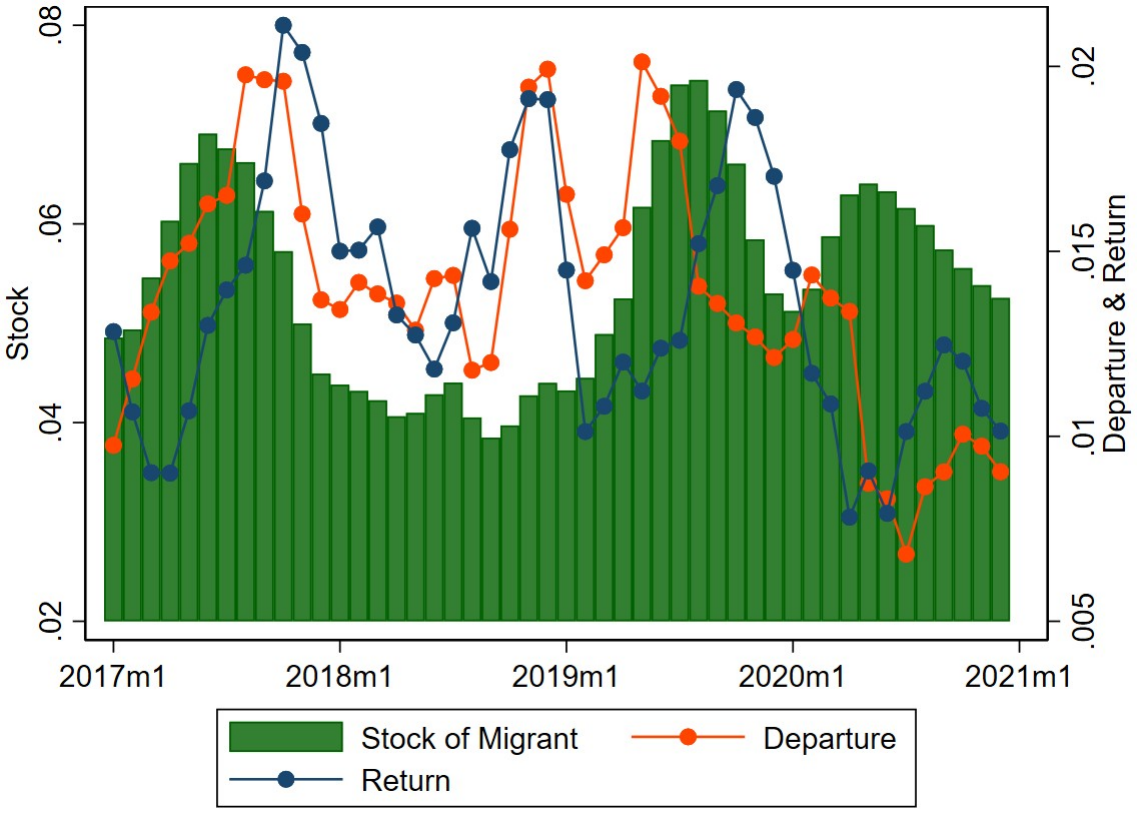
The impact of the COVID 19 on migration. This graph shows the stock, departure, and return of migrants, in terms of ratio to population, as a three-month moving average.

Fig 3 describes the stock, departure, and return of international migrants. The stock is defined as the (normalized) number of migrants as a proportion of the total population who are in the destination country in the month. The destination includes all countries, although most migrants are concentrated in Russia. Departure refers to the (normalized) number of migrants who have newly entered their destination countries in the month, and the return is the (normalized) number of migrants who have left their destination countries and returned to Tajikistan in the month. Both the departure and return are also normalized as a ratio to the total population. The stock in a month corresponds to the stock in the previous month, and adding the number of departures and subtracting the number of returns.

We observe a distinct seasonal pattern in the number of departures, returns and stock in regular years. The number of departures surpasses the number of returns in the first half of the year and the number of returns exceeds the number of departures in the second half. As a result, we see a seasonality to indicate that the stock of migrants in destination countries starts to increase in spring, stays high in summer, declines in autumn and reaches a low in winter. This pattern is obscured in 2018, which witnessed a large depreciation of the Russian ruble and a break in large-scale construction following the 2018 World Cup Soccer games, factors that hampered international migration.

We can see a different pattern in those numbers in 2020. The distinct feature of this year is that the numbers of both departures and returns are at a lower level compared to previous years. The number of departures declined in the first few months of 2020, contrasting to the regular increasing pattern observed in 2017 and 2019. The change in the trend is mostly a result of the border closures in mid-March 2020; many expecting migrants were forced to stay in the country because of the strict border closures and bans on international mobility. However, this is one side of a coin that captures inflow to the stock of migrants only. On the flip side, many migrants expecting to return to Tajikistan were not able to go back and were forced to stay in the destination countries, mostly in Russia, and thus the outflow of the stock of migrants was also affected. Indeed, the number of returns also declined after the closure. The number of returns bottomed out in spring and started to increase in subsequent months, and the number of departures that had lagged for a couple of months started to rebound in July.

The stock of Tajik migrants, which was high in 2019, has remained at a high level since the beginning of 2020. While both the numbers of departures and returns declined in 2020, the number of departures surpassed the number of returns in the first couple of months of 2020. As a result, we see a large stock of migrants in 2020 during the outbreak of the pandemic, despite the flow of migrants (i.e., the number of departures) being significantly reduced since April 2020. The large stock of migrants in 2020 might be counterintuitive but is well explained by the sharp decline in both numbers of departures and returns.

**Fig 4:**
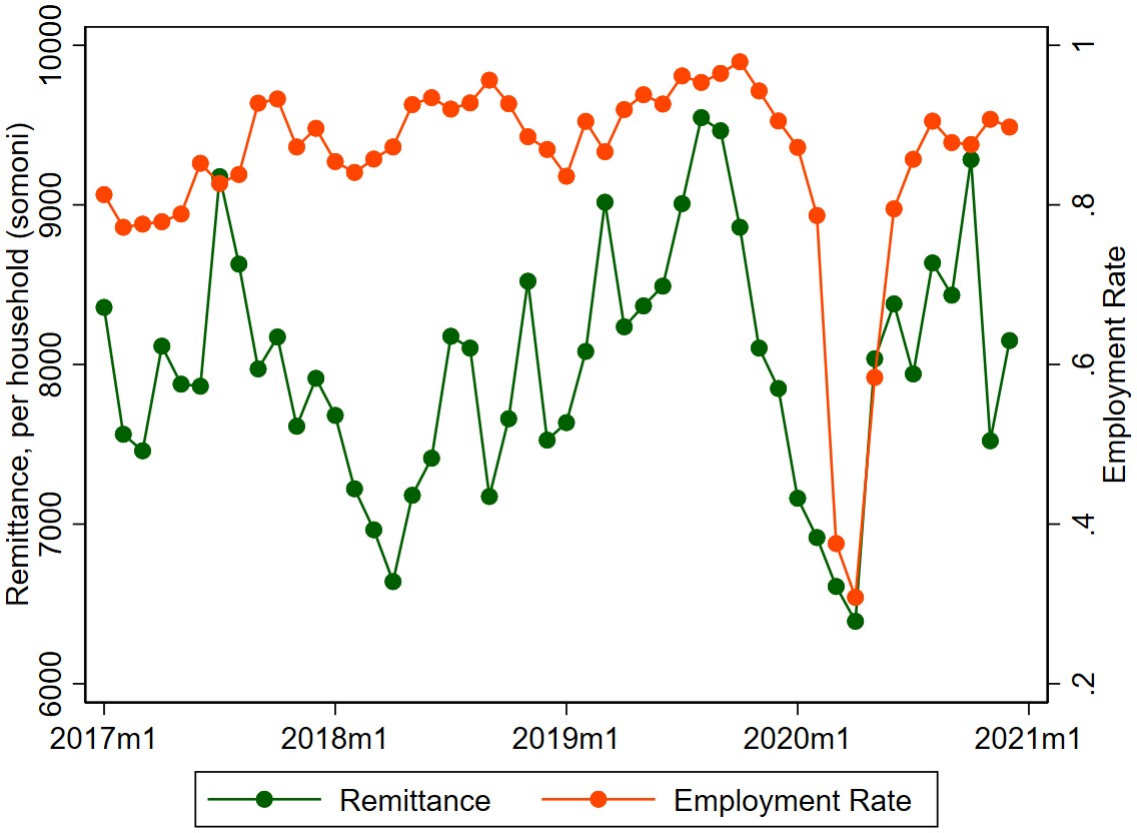
The impact of the COVID 19 on remittance and employment. This graph shows the remittance inflows and employment rate of migrants, in terms of ratio to population, using a three-month moving average.

One might argue that, even if the stock of migrants was not seriously affected by the pandemic, there might be a portion of migrants who were unemployed in their destination countries due to the COVID-19 pandemic, because the economy of destination countries was also seriously affected. Fig 4 describes the monthly employment rate of migrants and the monthly inflow of remittances, both of which are smoothed by taking a three-month moving average. The employment rate is defined as the proportion of migrants who were working in a survey month among the stock of migrants. The amount of remittances is calculated from survey responses asking how much remittances the household received in the previous ten days in Tajikistan somoni. The value is converted to a per household base dividing the total remittances by the number of households in the survey.

In regular years, the employment rate has been high and stable at around 80% to 90%. In 2020, we observe a sharp and large decline in the rate to a historically low level of around 30% in March and April, presumably because of the sharp economic downturn in Russia following the onset of the pandemic. However, we see a quick recovery of the employment rates of migrants from May, exceeding 80% in summer, which is by no means inferior to regular years. We see a large adverse shock on the employment rate caused by the pandemic in spring but the effect was only transitory.

The trend of the monthly remittance inflow is not much different from that of regular years, which fluctuates in a range between 7,000 to 9,000 somoni depending on the exchange rate and the seasonal change in the stock of migrants. In 2020, we see a large downside shock, with the remittance inflow declining substantially along with the substantial reduction in the employment rate. This was the case up until April, but we observe a rapid recovery from May to reach the regular level.

In sum, the impact of the COVID-19 pandemic on migration and remittances is sharp but transitory from March to May. Starting with a higher level at the beginning of 2020, the stock of migrants did not undergo a large decline because both departures and returns were reduced by the border closure and travel restriction. We see a sharp and adverse impact of the pandemic on the employment rate of migrants to a historically low level, but the employment rate quickly recovered to the same level as regular years in summer. The amount of remittance inflows substantially declined in March and April but rapidly regained their regular level in summer.

Therefore, we see that the impact of the pandemic on migration and remittance inflows was sharply detrimental to households in spring, but the effect was transitory, which conforms to the World Bank’s latest estimates on remittance inflow as of October [26]. They were revised upwardly compared with those as of April [27] and less pessimistic. However, the stock of international migrants was expected to decline in 2020 for the first time in recent history. Looking at Tajikistan, remittance inflows were expected to decrease to 2,066 million dollars in 2020 from 2,322 million dollars in 2019, an 11% decline, and the remittance inflows relative to GDP in 2020 were estimated to be 26.2%, only 2% points lower than 28.2% in 2019.

### Specification of the regression analyses

We examine the relationship between a variety of outcomes related to household welfare and household status of migration, remittance and employment in destination through the pandemic. The purpose of the regression analysis is to understand whether the households sending migrant and receiving remittances are better-off or not during the time of the COVID-19 pandemic. More precisely, we investigate whether there is any significant difference in the outcomes between households with migrants/remittances/employment in destination and those without them in the period after the onset of the COVID-19 pandemic.

We conduct an analysis to see how the implications of migration and remittances differ across months after April 2020 compared to regular years. For this purpose, we estimate the following equation:

$$y_{jt}=\sum_{C\in C\;}{\beta_{C}^{D}(D}_{jt}*C_{t})+\sum_{C\in C\;}{\gamma_{C}C}_{t}+\delta D_{jt}+\eta X_{jt}+Quarter_{t}+Year_{t}+\mu_{j}+\epsilon_{jt}$$ (1)

where *y*_*jt*_ is the outcome variable, *D*_*jt*_ is migration (or remittance or employment in destination) status of the household *j* in the quarter *t*. It takes 1 if the household *j* has a migrant member or receives remittances or has an employed in destination, and 0 otherwise. *C*_*t*_ indicates whether *t* is a quarter after the COVID-19 pandemic began where *C* is an element of the set ℂ such that

$$C\in \left\{C:April-June20,\;July-September20,\;October-December20\right\}$$

For example, *April* − *June*20_*t*_ takes 1 if *t* is April-June 2020, and it takes 0 otherwise. *γ*_*C*_ captures overall impact specific to quarter *C* in 2020, conditional on the year trend and seasonality captured by the data before 2019. $\beta_{C}^{D}$ then represents the differential impact of migration or remittances or employment in destination in the quarter of *C* since April 2020. *D*_*jt*_ is the migration (or remittance or employment in destination) status of the household *j* in the quarter *t*. *X*_*jt*_ is a vector of covariates that may affect the outcome variables. We include the incidence of disruption in the electricity and water supply services in the previous month because these can be a source of noise for the well-being of the household. *Year*_*t*_ and *Quarter*_*t*_ are the year and month fixed effect, respectively. *μ*_*j*_ is the household fixed effect, which addresses time-invariant characteristics of each household that can be correlated with both migration and remittance decisions as well as the outcomes. Household-level fixed effect estimations are implemented by a within-estimator by subtracting household-level means of all variables from each observation. While the dependent variables are binary we do not employ the methods for limited dependent variables such as logit and probit, since it is difficult to interpret the estimated coefficients of such non-linear models if the variable(s) of interest is the interaction term(s) like in our case [28]. In our linear probability model, we can interpret the coefficients as the mean difference of the outcome variable between the group whose interaction term is one and the group with zero, conditional on other covariates and fixed effects.

This empirical specification is designed to detect any significant changes in seasonality in 2020. The coefficients $\beta_{C}^{D}$ in Eq (1) are properly identified if the interaction term (*D*_*jt*_ * *C*_*t*_) is not correlated with the unobservable. This condition can be violated in several ways. First, the time-invariant characteristics of each household can be correlated with both migration and remittance decisions as well as the outcomes. This is addressed in Eq (1) by introducing the household fixed effect, *μ*_*j*_. The second threat is reverse causality. The key motivation for sending migrants is to improve household economic welfare in their home country through remittances. Hence, changes in migration status and remittances can be the consequences of household welfare outcomes. Lastly, there could be regional/industry-specific shocks that may simultaneously affect migration/remittance and household outcomes.

In order to address these identification concerns, we confine the analysis on a subset of the households that sent migrants in 2019, in addition to the introduction of household fixed effect. These households are more likely to rely on migration compared to those that have never sent migrants during the study period since 2015. Among those households dependent on migration and remittances, reverse causality and selection bias of estimation should be much less serious, because they should share similar unobserved characteristics affecting migration.

## Results and discussion of the regression analyses

Table 3 presents the estimation results on the impact of migrants. First, we observe overall negative shock on a variety of welfare outcomes of Tajik households since the COVID-19 pandemic started, which is shown by the coefficients on *April-June2020*, *July–September2020*, and *October-December2020*. Column (1) shows that the probability of households being able to buy enough food has been significantly lower in all those quarters. The coefficients are positive and significant in Column (2), showing a significantly higher probability that households were not able to buy enough food for children in the same period. Under the “with-COVID-19” period since April 2020, households underwent significantly unfavorable conditions in being unable to pay for utilities (Column (3)) and increasing borrowing to meet daily needs (Column (4)). However, in most cases, we do not see significant coefficients in health spending (Column (5)) or employment (Columns (6) and (7)). The subjective perceptions of financial and economic wellbeing are also significantly worse and are reported in Columns (8) to (12).

**Table 3:** Impact of migration on household welfare during the with-COVID-19 period.

VARIABLES	(1)	(2)	(3)	(4)	(5)	(6)	(7)	(8)	(9)	(10)	(11)	(12)
	Able to buy enough food for members	Not able to buy enough food for the children	Financially unable to pay for utilities	Borrow any money to pay for basic needs	Reduced healthcare expenditure	Did work for pay in 7 days	Received wage in 10 days	Perceives own HH as poor	Living area’s economic condition is bad	Area’s job situation is bad	Current financial condition is worse	Financial condition gets worse
Migrate X APR_JUN20	-0.0207	0.0190	-0.0467*	-0.0192	-0.0287	-0.00754	-0.0549***	-0.0750***	-0.0265	-0.0836***	-0.0281	0.00644
	(0.0161)	(0.0124)	(0.0252)	(0.0247)	(0.0488)	(0.0243)	(0.0161)	(0.0245)	(0.0215)	(0.0284)	(0.0208)	(0.0137)
Migrate X JUL_SEP20	-0.00337	-0.0176	-0.0430*	-0.0213	-0.0122	-0.0346	-0.0123	-0.0931***	-0.0445**	-0.0806***	-0.0346*	-0.00836
	(0.0149)	(0.0117)	(0.0227)	(0.0221)	(0.0488)	(0.0228)	(0.0176)	(0.0237)	(0.0187)	(0.0270)	(0.0179)	(0.0107)
Migrate X OCT_DEC20	-0.0134	-0.0175*	-0.0322	-0.0338	-0.0204	-0.0363	-0.0137	-0.101***	-0.0253	-0.0578**	0.0139	0.0116
	(0.0127)	(0.00965)	(0.0205)	(0.0224)	(0.0585)	(0.0247)	(0.0163)	(0.0264)	(0.0234)	(0.0292)	(0.0170)	(0.0109)
APR_JUN20	-0.0293**	0.0195*	0.115***	0.0758***	0.0208	-0.105***	0.0173	0.0986***	0.0882***	0.216***	0.123***	0.0238**
	(0.0144)	(0.0115)	(0.0223)	(0.0202)	(0.0418)	(0.0218)	(0.0135)	(0.0211)	(0.0188)	(0.0234)	(0.0187)	(0.0119)
JUL_SEP20	-0.0574***	0.0434***	0.119***	0.0531***	0.0256	-0.00352	0.00208	0.0385*	0.0755***	0.265***	0.132***	0.0478***
	(0.0137)	(0.0120)	(0.0206)	(0.0192)	(0.0429)	(0.0200)	(0.0163)	(0.0207)	(0.0174)	(0.0236)	(0.0164)	(0.0103)
OCT_DEC20	-0.0237**	0.0240**	0.0391**	0.0331*	0.0547	-0.00860	-0.00168	0.0367*	0.131***	0.112***	0.107***	0.0411***
	(0.0118)	(0.00937)	(0.0192)	(0.0197)	(0.0453)	(0.0198)	(0.0151)	(0.0221)	(0.0186)	(0.0239)	(0.0141)	(0.00948)
Electricity outage	-0.0337***	0.0277***	0.0806***	0.0639***	0.0481***	-0.0161***	0.0119***	0.0227***	0.0491***	0.0689***	0.0401***	0.0302***
	(0.00489)	(0.00419)	(0.00631)	(0.00605)	(0.00910)	(0.00560)	(0.00401)	(0.00643)	(0.00533)	(0.00697)	(0.00419)	(0.00334)
Water disruption	-0.0464***	0.0262***	0.0911***	0.0581***	0.0746***	-0.0397***	0.0125*	0.0615***	0.0308***	0.0212*	0.0356***	0.0199***
	(0.00882)	(0.00803)	(0.0110)	(0.0108)	(0.0134)	(0.00887)	(0.00700)	(0.0107)	(0.00882)	(0.0118)	(0.00785)	(0.00674)
Migrant member in this month	0.0195***	0.000706	0.0306***	0.0202**	0.00475	-0.115***	-0.00165	0.0295***	0.00168	0.0563***	-0.0111**	-0.00824**
	(0.00641)	(0.00439)	(0.00828)	(0.00792)	(0.0123)	(0.00876)	(0.00561)	(0.00921)	(0.00655)	(0.00986)	(0.00481)	(0.00409)
Observations	31,223	26,221	31,223	31,223	13,032	31,223	31,223	28,865	28,865	28,865	31,223	31,223
R-squared	0.105	0.031	0.071	0.030	0.043	0.051	0.012	0.053	0.019	0.042	0.116	0.116
Number of households	773	763	773	773	739	773	773	770	770	770	773	773

*Source*: Authors.

*Note*: Standard errors are clustered at the level of household. Other explanatory variables not shown in this table are year dummy, quarter dummy, Migration Status, electricity outage, and water disruption. Household-level fixed effect estimation are implemented by a within-estimator to subtract household-level means of variables from each observation.

*** p<0.01,

** p<0.05,

* p<0.1.

Second, despite these overall negative impacts during the with-COVID-19 period, households with migrants are in general better-off for some outcomes. Although most of the coefficients are not statistically significant, households with migrants are less likely to fail in supplying enough food for family members and children (Columns (1) and (2)), paying for utilities in April to September (Column (3)) and borrowing (not significant in Column (4)). They were more likely to avoid reducing healthcare expenditure (not significant in Column (8)). Furthermore, households with migrants are less pessimistic during this hard time. Compared to non-migrant households, migrant households are significantly less inclined to answer that they are poor (Column (8)), that the economic condition in living area is bad (Column (9)), that area’s job situation is bad (Column (10)), that the financial situation is becoming worse (Column (11)) and that the economy of the area they live will further deteriorate (not significant in Column (12)).

This resilience of migrant households seems to stem from the remittances they continue receiving even after COVID-19 severely hit Russia and the border closed in March. Table 4 reports the estimation results on the impact of remittances. We observe that coefficients on the quarter dummies in 2020 in a variety of welfare outcomes in Columns (1) to (4) imply that these outcomes worsened during the months after April 2020. At the same time, most of the coefficients on the interaction term of remittances and quarters are negative, and in some cases, significant in the period of April to June in Columns (1) to (3), suggesting that the overall unfavorable shocks on these outcome variables are mitigated for households receiving remittances compared to households not receiving remittances. While most of the coefficients on the quarter dummies in 2020 are not significant in Column (5) to (7), the coefficients on the interaction terms are negative and significant in July to September (Column (5)) and July to September and October to December (Column (6)), showing that households receiving remittances are less likely to reduce health spending and more likely to work for pay in the previous seven days.

**Table 4:** Impact of remittances on household welfare during the with-COVID-19 period.

VARIABLES	(1)	(2)	(3)	(4)	(5)	(6)	(7)	(8)	(9)	(10)	(11)	(12)
	Able to buy enough food for members	Not able to buy enough food for the children	Financially unable to pay for utilities	Borrow any money to pay for basic needs	Reduced healthcare expenditure	Did work for pay in 7 days	Received wage in 10 days	Perceives own HH as poor	Living area’s economic condition is bad	Area’s job situation is bad	Current financial condition is worse	Financial condition gets worse
Remittance X_APR_JUN20	0.0402***	-0.0250**	-0.0773***	-0.0313	-0.0431	-0.0177	-0.00119	-0.102***	-0.0640***	-0.119***	-0.108***	-0.0287*
	(0.0141)	(0.0127)	(0.0257)	(0.0275)	(0.0586)	(0.0309)	(0.0198)	(0.0277)	(0.0201)	(0.0333)	(0.0188)	(0.0150)
Remittance X_JUL_SEP20	0.00461	-0.0112	-0.0225	-0.0356	-0.0831**	-0.0521**	0.0241	-0.129***	-0.0180	-0.0970***	-0.0480***	-0.0106
	(0.0143)	(0.0125)	(0.0225)	(0.0222)	(0.0387)	(0.0228)	(0.0155)	(0.0247)	(0.0161)	(0.0292)	(0.0161)	(0.0106)
Remittance X_OCT_DEC20	-0.0173	0.00589	-0.0330	-0.0114	-0.0575	-0.0464*	-0.0184	-0.154***	-0.0562**	-0.0165	0.0118	0.0146
	(0.0130)	(0.00959)	(0.0211)	(0.0237)	(0.0682)	(0.0261)	(0.0164)	(0.0263)	(0.0224)	(0.0295)	(0.0181)	(0.0128)
APR_JUN20	-0.0468***	0.0339***	0.0958***	0.0680***	0.00731	-0.107***	-0.0158	0.0659***	0.0801***	0.180***	0.119***	0.0312***
	(0.0113)	(0.00949)	(0.0155)	(0.0145)	(0.0306)	(0.0164)	(0.0101)	(0.0158)	(0.0135)	(0.0166)	(0.0130)	(0.00877)
JUL_SEP20	-0.0605***	0.0355***	0.0981***	0.0480***	0.0386	-0.0128	-0.0105	0.0108	0.0531***	0.238***	0.122***	0.0452***
	(0.0110)	(0.00979)	(0.0154)	(0.0150)	(0.0342)	(0.0163)	(0.0121)	(0.0165)	(0.0130)	(0.0184)	(0.0119)	(0.00787)
OCT_DEC20	-0.0275***	0.0135*	0.0281*	0.0169	0.0565	-0.0189	-0.00550	0.0129	0.128***	0.0835***	0.112***	0.0445***
	(0.0105)	(0.00815)	(0.0153)	(0.0159)	(0.0373)	(0.0165)	(0.0119)	(0.0178)	(0.0141)	(0.0189)	(0.0106)	(0.00741)
Electricity outage	-0.0338***	0.0278***	0.0808***	0.0640***	0.0482***	-0.0160***	0.0119***	0.0229***	0.0492***	0.0691***	0.0402***	0.0302***
	(0.00489)	(0.00418)	(0.00632)	(0.00605)	(0.00910)	(0.00560)	(0.00401)	(0.00643)	(0.00534)	(0.00696)	(0.00418)	(0.00334)
Water disruption	-0.0464***	0.0263***	0.0912***	0.0582***	0.0745***	-0.0396***	0.0125*	0.0617***	0.0309***	0.0213*	0.0356***	0.0199***
	(0.00882)	(0.00803)	(0.0110)	(0.0108)	(0.0134)	(0.00887)	(0.00701)	(0.0107)	(0.00882)	(0.0118)	(0.00784)	(0.00674)
Received remittance in 10 days	0.0174***	0.000158	0.0261***	0.0175**	0.00456	-0.117***	-0.00589	0.0208**	-0.00159	0.0473***	-0.0120**	-0.00755**
	(0.00589)	(0.00408)	(0.00796)	(0.00755)	(0.0118)	(0.00838)	(0.00521)	(0.00896)	(0.00653)	(0.00938)	(0.00485)	(0.00379)
Observations	31,223	26,221	31,223	31,223	13,032	31,223	31,223	28,865	28,865	28,865	31,223	31,223
R-squared	0.105	0.031	0.071	0.030	0.044	0.051	0.012	0.055	0.019	0.042	0.117	0.116
Number of households	773	763	773	773	739	773	773	770	770	770	773	773

*Source*: Authors.

*Note*: Standard errors are clustered at the level of household. Other explanatory variables not shown in this table are year dummy, quarter dummy, Migration Status, electricity outage, and water disruption. Household-level fixed effect estimation are implemented by a within-estimator to subtract household-level means of variables from each observation.

*** p<0.01,

** p<0.05,

* p<0.1.

The same argument also applies to the subjective perception of financial and economic wellbeing in Columns (8) to (12). Compared to non-receiving households, remittance-receiving households during the time of COVID-19 are less likely to think they are poor (Column (8)) and they are more optimistic about the economic and employment situation of their living area (Columns (9) and (10)). They are less pessimistic about current and future financial conditions (Columns (11) and (12)). These results are consistent with UNDP (2020), showing that remittances continue to play a significant role in consumption.

The same pattern on the coefficients is also observed when we examine the impact of employment in destination. Table 5 reports the estimation results on the impact of employment in destination. The coefficients on the quarter dummies in 2020 in Columns (1) to (4) show that these outcomes worsened after the outbreak of the pandemic. At the same time, most of the coefficients on the interaction term of remittances and quarters imply that the negative impact was abbreviated (significant in some cases), suggesting that the overall unfavorable shocks on these outcome variables are mitigated for households by being employed in destination. Most of the coefficients on the quarter dummies in 2020 are not significant in Column (5) to (7), the coefficients on the interaction terms are negative and significant in July to September (Column (6)) and April to June and July to September (Column (7)), showing that households with employment at a migration destination are less likely to work for pay and receive wages. The pattern on the subjective perception of financial and economic wellbeing in Columns (8) to (12) is similar to those in Table 4. Compared to households without employment at a migration destination, households with employment at a destination during the time of COVID-19 are less likely to think they are poor (Column (8)), that they are more optimistic about the economic and employment situation of their living area (Columns (9) and (10)), and that they are less pessimistic about current and future financial conditions (Columns (11) and (12)).

**Table 5:** Impact of employment at migration destinations on household welfare during the with-COVID-19 period.

VARIABLES	(1)	(2)	(3)	(4)	(5)	(6)	(7)	(8)	(9)	(10)	(11)	(12)
	Able to buy enough food for members	Not able to buy enough food for the children	Financially unable to pay for utilities	Borrow any money to pay for basic needs	Reduced healthcare expenditure	Did work for pay in 7 days	Received wage in 10 days	Perceives own HH as poor	Living area’s economic condition is bad	Area’s job situation is bad	Current financial condition is worse	Financial condition gets worse
Employed X APR_JUN20	0.0387*	-0.0260	-0.0667*	-0.0161	-0.0649	-0.00193	-0.0497***	-0.0748**	-0.0522*	-0.0846*	-0.0468*	-0.0239
	(0.0201)	(0.0165)	(0.0348)	(0.0363)	(0.0830)	(0.0337)	(0.0175)	(0.0354)	(0.0308)	(0.0495)	(0.0273)	(0.0169)
Employed X JUL_SEP20	-0.00340	-0.0176	0.0100	-0.00389	-0.00744	-0.0847**	-0.0487***	-0.0824**	-0.0291	-0.0882**	-0.0255	-0.0345***
	(0.0207)	(0.0155)	(0.0316)	(0.0333)	(0.0692)	(0.0350)	(0.0142)	(0.0328)	(0.0232)	(0.0394)	(0.0202)	(0.0105)
Employed X OCT_DEC20	-0.0195	-0.00672	-0.0177	-0.00214	0.0204	-0.0348	-0.00403	-0.0697*	-0.0563*	-0.0753*	-0.0418**	-0.00238
	(0.0198)	(0.0138)	(0.0288)	(0.0337)	(0.0923)	(0.0363)	(0.0192)	(0.0394)	(0.0305)	(0.0412)	(0.0203)	(0.0154)
APR_JUN20	-0.0448***	0.0328***	0.0913***	0.0653***	0.00597	-0.109***	-0.0121	0.0588***	0.0761***	0.172***	0.110***	0.0295***
	(0.0112)	(0.00937)	(0.0153)	(0.0146)	(0.0294)	(0.0164)	(0.0104)	(0.0154)	(0.0131)	(0.0163)	(0.0126)	(0.00862)
JUL_SEP20	-0.0591***	0.0355***	0.0919***	0.0408***	0.0191	-0.0126	0.00115	-0.00586	0.0531***	0.229***	0.115***	0.0476***
	(0.0108)	(0.00964)	(0.0149)	(0.0146)	(0.0315)	(0.0157)	(0.0121)	(0.0163)	(0.0128)	(0.0183)	(0.0116)	(0.00768)
OCT_DEC20	-0.0290***	0.0154*	0.0233	0.0148	0.0419	-0.0247	-0.00882	-0.0113	0.123***	0.0881***	0.119***	0.0477***
	(0.0104)	(0.00824)	(0.0149)	(0.0157)	(0.0354)	(0.0164)	(0.0116)	(0.0170)	(0.0137)	(0.0183)	(0.0106)	(0.00741)
Electricity outage	-0.0337***	0.0278***	0.0807***	0.0640***	0.0480***	-0.0159***	0.0119***	0.0231***	0.0493***	0.0692***	0.0401***	0.0302***
	(0.00489)	(0.00418)	(0.00631)	(0.00605)	(0.00911)	(0.00560)	(0.00401)	(0.00644)	(0.00535)	(0.00697)	(0.00419)	(0.00334)
Water disruption	-0.0464***	0.0263***	0.0913***	0.0582***	0.0746***	-0.0396***	0.0125*	0.0617***	0.0310***	0.0214*	0.0357***	0.0199***
	(0.00881)	(0.00803)	(0.0110)	(0.0108)	(0.0134)	(0.00888)	(0.00701)	(0.0107)	(0.00882)	(0.0118)	(0.00785)	(0.00675)
Migrant member employed	0.0175***	0.000161	0.0247***	0.0165**	0.00276	-0.118***	-0.00515	0.0163*	-0.00266	0.0458***	-0.0129***	-0.00732*
	(0.00585)	(0.00405)	(0.00794)	(0.00751)	(0.0117)	(0.00835)	(0.00524)	(0.00893)	(0.00656)	(0.00946)	(0.00485)	(0.00374)
Observations	31,223	26,221	31,223	31,223	13,032	31,223	31,223	28,865	28,865	28,865	31,223	31,223
R-squared	0.105	0.031	0.071	0.030	0.043	0.051	0.012	0.052	0.019	0.042	0.116	0.116
Number of households	773	763	773	773	739	773	773	770	770	770	773	773

*Source*: Authors.

*Note*: Standard errors are clustered at the level of household. Other explanatory variables not shown in this table are year dummy, quarter dummy, Migration Status, electricity outage, and water disruption. Household-level fixed effect estimation are implemented by a within-estimator to subtract household-level means of variables from each observation.

*** p<0.01,

** p<0.05,

* p<0.1.

## Conclusion

This paper examines the impact of the COVID-19 pandemic on a variety of economic outcomes on household welfare by using a unique high-frequency household panel dataset that covers the period both before and after the outbreak. We provide several important findings. First, the adverse effect of the pandemic was severe in the second quarter of the 2020s. Second, contrasting to expectation, the pandemic had a sharp but only transitory effect on the number of migrants temporarily staying abroad. A portion of planned migrants could not make their departure to the destination country (mainly the Russian Federation) under the border closures, while some of the migrants expecting to return were also stuck in the destination country. Third, despite a sharp decline in employment and the remittances of migrants in April and May, both factors quickly recovered. Regression analyses confirmed migration and remittances and employment at the destination had eased the economic shock of the COVID-19 pandemic in terms of not only material well-being such as food, utilities, consumption, and health but also their views on their financial and economic situations.

These findings show that the unfavorable effect of the COVID-19 pandemic was severe and temporary right after the outbreak, but households with migrants were more resilient against the pandemic, which is in accordance with other existing studies showing that migration and remittances serve as a form of insurance. These findings have implications for policymakers concerning who should be prioritized for government support in times of economic crisis. Our results indicate that households who are not receiving remittances may be less resilient during periods of crisis, and governments should target groups with fewer opportunities for migration and allocate resources for relief against crisis appropriately. The contribution of this paper to the literature is to provide empirical evidence on how welfare of migrant households responds to an economy-wide shock, taking the COVID-19 pandemic as a case. In addition, we utilize a monthly panel dataset covering the period before and after the outbreak of the pandemic to provide a more granular picture on the impact of the COVID-19 pandemic on migration and remittances in developing countries.

While this study contributes to deepening our understanding of the short-term economic impacts of the COVID-19 pandemic, it is still too early to conclude what the mid- and long-term consequences may be. This paper shows that the impacts change over time and can even flip within this short period of time. In addition, there are various anecdotal accounts suggesting that many migrants have been stranded at the airports or have been working under unfavorable hygienic condition without sufficient access to healthcare services in the destination countries [29]. Some other migrants have trouble sending remittances due to the lack of access to digital means for remittances. These potential costs and burdens on migrants and their families during this COVID-19 time are not explicitly assessed in this study. We thus need to undertake further studies to collect data over a longer period to understand the impacts of the pandemic more comprehensively.

## Supporting information

S1 Appendix Seasonal patterns of household welfare. (DOCX)
