# Supplementary material for: Resilience against the pandemic: The impact of COVID-19 on migration and household welfare in Tajikistan
Source: PLoS One. 2021 Sep 20;16(9):e0257469. doi: 10.1371/journal.pone.0257469 (PMC8452056; doi:10.1371/journal.pone.0257469)
Supplement: S1 Appendix — (DOCX) [file pone.0257469.s001.docx]

# S1 Appendix to;

**Resilience against the pandemic:**

**the impact of COVID-19 on migration and household welfare in Tajikistan**

Satoshi Shimizutani* and Eiji Yamada^[[1]](#footnote-1)^

# Seasonal patterns of household welfare

This appendix describes the impact of the COVID-19 pandemic on a variety of household welfare outcomes by comparing the seasonal patterns in between “regular” years without the pandemic and 2020 with the pandemic. We define “regular” years as the average of 2018 and 2019. We limit the regular years to 2018 and 2019 because some variables have been collected only after the middle of 2017. In all figures, we standardize the value of January as one and depict seasonal movements in subsequent months by contrasting households with migrants to households without migrants using weights to each household. Thus, each graph has four lines. We take three-month moving average for all series to make the seasonal pattern clear.

Food security

The first two graphs report the proportion of households that were able to buy enough food for members for the previous ten days and that were not able to buy enough food for the children in the same period. We see a decline of the share of households able to buy enough food for members in a parallel way to April and the share recovered faster for households with migrants. This is also the case for the proportion of households unable to buy enough food for the children. The share increased for both households and peaked out earlier for migrant-sending households in April, with a faster decline for those households up to the level observed in regular years. In contrast, the proportion declined slowly for households without migrants. We also examined other right measures on food security measures in the survey and observed a similar pattern: food security worsened at the same pace or faster for households with migrants and recovered faster for them, while the current level is not worse in most cases (not shown).

| 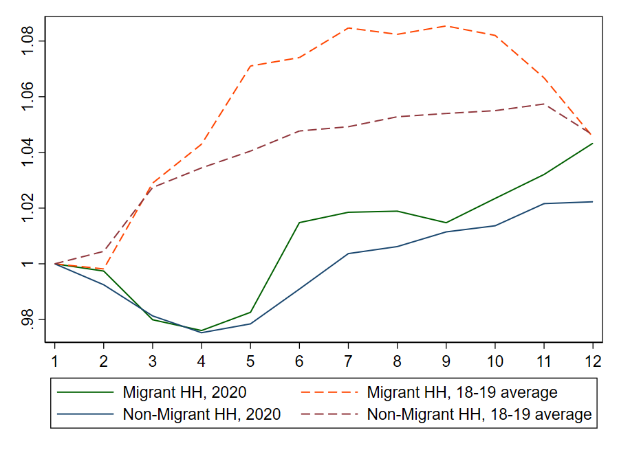 | 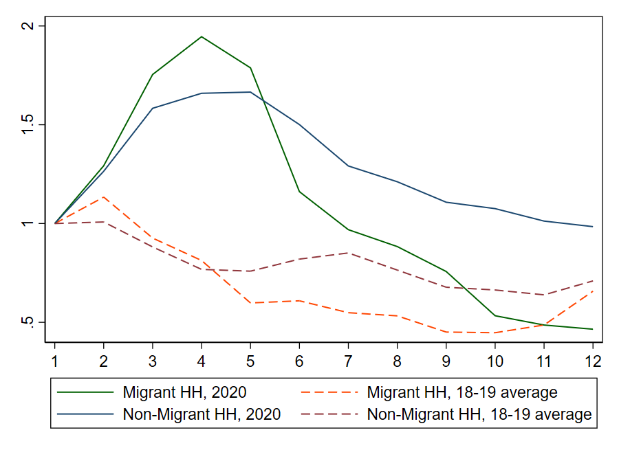 |
| --- | --- |
| **Fig A1. Food Security: Able to buy enough food** | **Fig 2B. Food Security: Not able to buy enough food for children** |

Finance for basic needs

The left-hand side graph (Fig A3) shows the proportion of households financially unable to pay for utilities in the previous ten days. A rapid increase in 2020 is observed for both households with and without migrants up to May but the share peaked out earlier and declined faster for households with migrants to the level in regular years. The right-hand side reports the share of households borrowing any money to pay for basic needs over the previous ten days increased in spring at a faster rate for households with migrants. At a glance, migrant-sending households were worse off than households without migrants, but we note that those households had fewer liquidity constraints and found it easier to borrow than non-migrant households. By autumn, the proportion was returning to the regular pattern in both households with and without migrants. The pattern of households toward borrowing is similar to that of households selling assets to pay for basic needs (not shown).

| 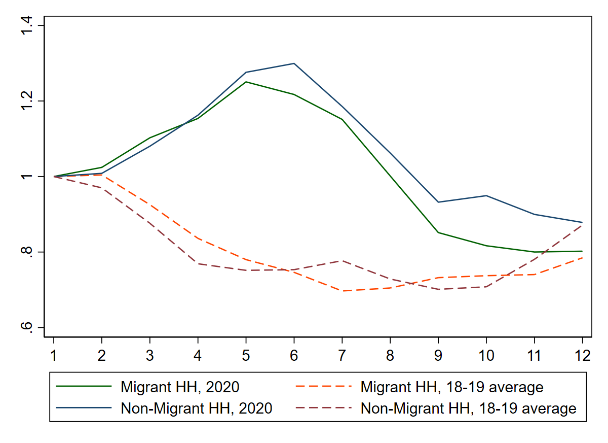 | 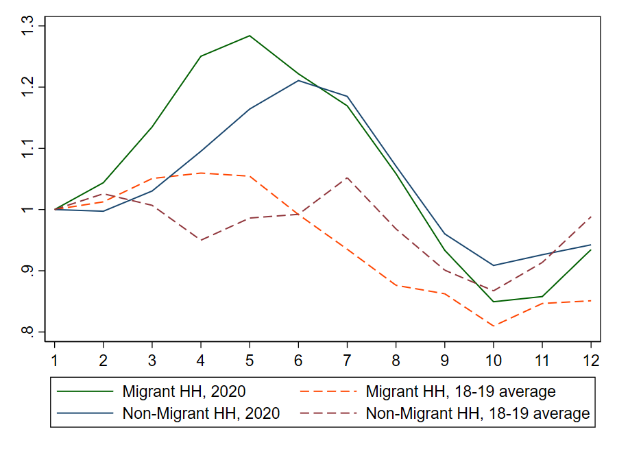 |
| --- | --- |
| **Fig A3. Financing for basic needs: Unable to pay for utilities** | **Fig A4. Financing for basic needs: Borrowed money for basic needs** |

Healthcare expenditure

The graph shows the proportion of households that reduced health expenditure. It shows a gradual decline in subsequent months for both households with and without migrants and there is no serious effect from the COVID-19 pandemic on use of health care and no distinction is observed between households with and without migrants. The proportion of households with any member who has been sick over the previous ten days and tracks the regular seasonal pattern in both migrant and non-migrant households (not shown). We do not see any different seasonal pattern in 2020 under the pandemic.

| 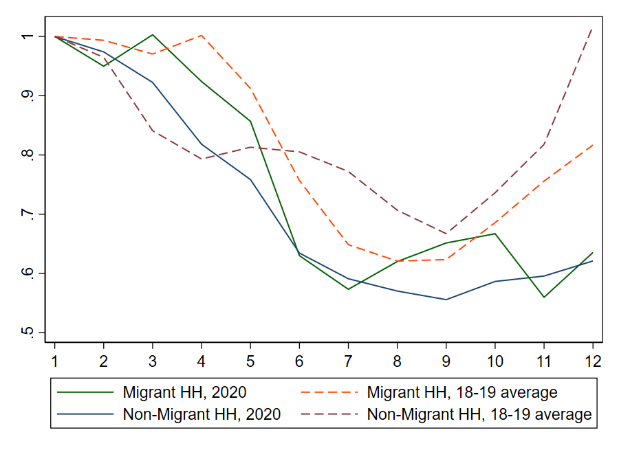 |
| --- |
| **Fig A5. Reduce healthcare expenditure** |

Employment

The first graph shows the proportion of households that did any paid work in the previous seven days. Reflecting the economic downturn during the pandemic, the proportion started to increase from April at a slower speed for households with migrants and was lower for households without migrants in spring. The proportion of households with migrants started to catch up to the regular pattern in autumn, while that for households without migrants bottomed out in May. The second graph shows the proportion of households that received a wage in the previous ten days. The proportion declined sharply for households with migrants and bottomed out in June. The proportion for households without migrants is not worse compared to regular years in autumn but a further decline is observed after September in households both with and without migrants.

| 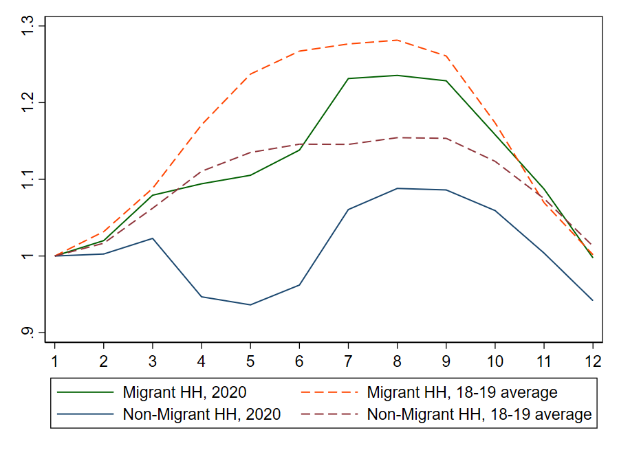 | 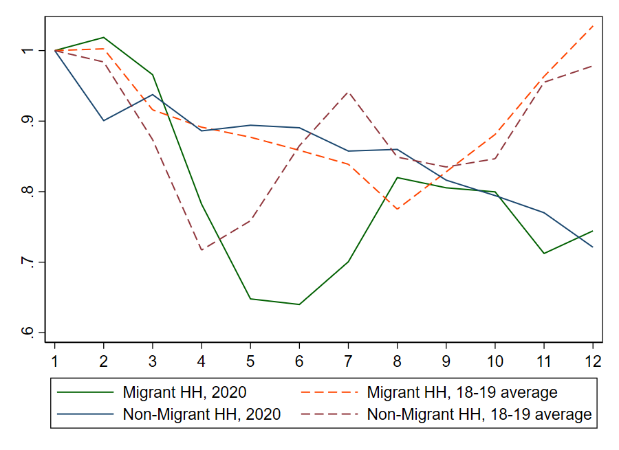 |
| --- | --- |
| **Fig A6. Employment: Did any paid work** | **Fig A7. Employment: Received wage** |

Subjective economic wellbeing

There are three outcomes. First, the proportion of households that think the economic wellbeing of their household is poor started to increase in households both with and without migrants, contrasting with the decline in regular years, while households with migrants peaked out earlier in April, converting to the regular seasonal pattern in autumn. Second, there is a surge in the proportion of households that believe the current economic conditions in the city or area where they live are bad or very bad also started to increase in households both with and without migrants which peaked out earlier for those with migrants and declined up to September. We observe a surge of the proportion in households both with and without migrants after September. Third, the proportion of households that think now is a bad time to find a job in the city or area where they live today was getting worse up to the summer, but then peaked out with a small increase after October.

| 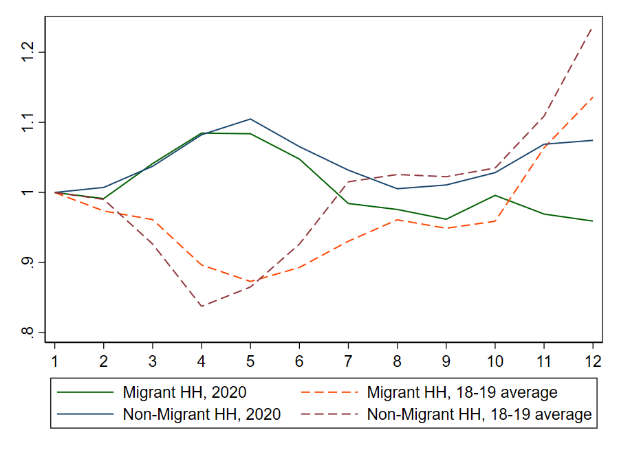 | 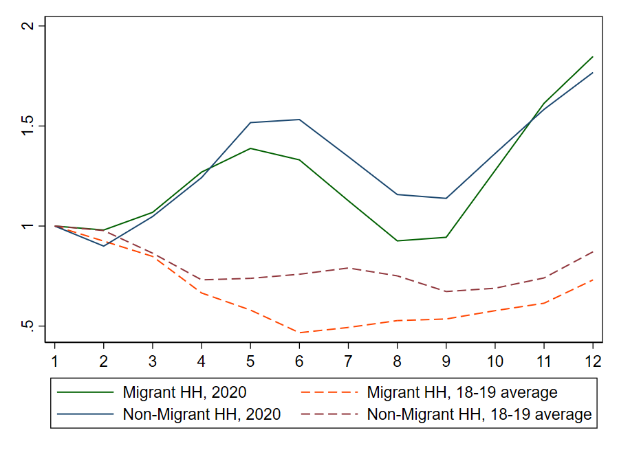 |
| --- | --- |
| **Fig A8. Subjective economic wellbeing: Perceive own household as poor** | **Fig A9. Subjective economic wellbeing: Area’s economic condition is bad** |
| 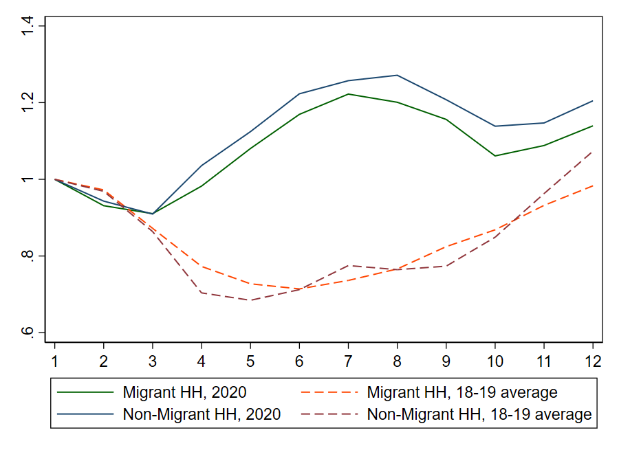 |  |
| **Fig A10. Subjective economic wellbeing: Area’s job situation is bad** |  |

Financial Wellbeing

The proportion of households thinking that the current financial condition is worse than the previous month (left-hand side) started to expand rapidly in 2020, compared to a gradual decline month-by-month in regular years. The share increased three-fold in April relative to January for both households with and without migrants, but we observe the proportion peaked out in May for households with migrants earlier than households without migrants in June. We note that the proportion leveled off in summer but started increase again after September. On the other hand, the proportion of households expecting financial conditions to get worse in the next month (right-hand side) is higher for migrant households; the share peaks out in April for them and declines faster than non-migrant households. These observations suggest that, under the pandemic, the financial wellbeing of households with migrants at present got out of the worst earlier. They also had greater anxiety about the future at the onset but this pessimistic view was weak by early summer. However, we should pay attention to the cessation in decline in autumn.

| 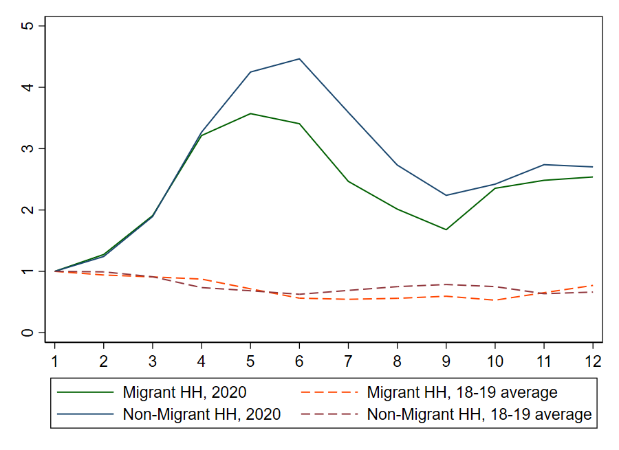 | 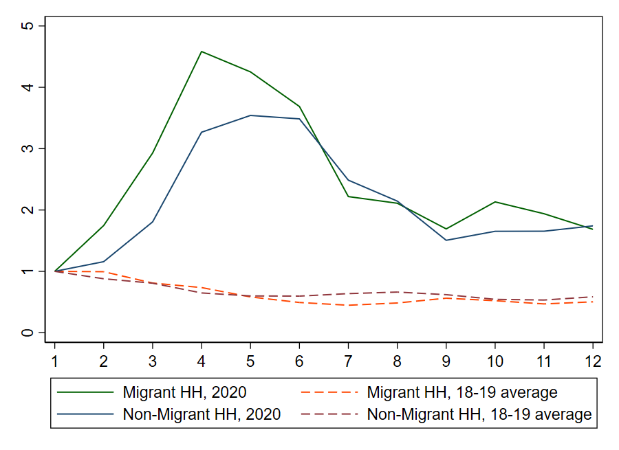 |
| --- | --- |
| **Fig A11. Financial wellbeing: Financial condition is worse** | **Fig A12. Financial wellbeing: Financial condition will become worse** |

Those simple calculations show that the impact of the COVID-19 pandemic was temporarily serious in terms of economic measures in March to May, but it seems that households with migrants emerged from their worst difficulties earlier and have been recovering to meet the regular seasonal pattern. Households without migrants are also returning to the regular pattern, but lagging behind households with migrants. On the other hand, we do not see any distinct difference in health status or medical care. We observe a severe drop in household welfare in spring, as the previous studies confirmed. What is newly found in this study is that household welfare measured in a variety of outcomes is now normalizing. These observations may be counterintuitive, since many of those expecting to migrate were forced to stay in their home country due to travel restrictions and border closures, which would be plausibly detrimental to remittance inflows this year.

1. JICA Ogata Sadako Research Institute for Peace and Development, Tokyo, Japan.

   * Corresponding author. E-mail: [Shimizutani.Satoshi@jica.go.jp](mailto:Shimizutani.Satoshi@jica.go.jp) [↑](#footnote-ref-1)
